# On the Use of Machine Learning Techniques and Non-Invasive Indicators for Classifying and Predicting Cardiac Disorders

**DOI:** 10.3390/biomedicines11102604

**Published:** 2023-09-22

**Authors:** Raydonal Ospina, Adenice G. O. Ferreira, Hélio M. de Oliveira, Víctor Leiva, Cecilia Castro

**Affiliations:** 1 Department of Statistics, Universidade Federal da Bahia, Salvador 40110-909, Brazil; 2 Department of Statistics, CASTLab, Universidade Federal de Pernambuco, Recife 50670-901, Brazil; 3 School of Industrial Engineering, Pontificia Universidad Católica de Valparaíso, Valparaíso 2362807, Chile; 4 Centre of Mathematics, Universidade do Minho, 4710-057 Braga, Portugal

**Keywords:** biological indicators, cardiopathy, classification models, data science, machine learning, resource efficiency

## Abstract

This research aims to enhance the classification and prediction of ischemic heart diseases using machine learning techniques, with a focus on resource efficiency and clinical applicability. Specifically, we introduce novel non-invasive indicators known as Campello de Souza features, which require only a tensiometer and a clock for data collection. These features were evaluated using a comprehensive dataset of heart disease cases from a machine learning data repository. Our findings highlight the ability of machine learning algorithms to not only streamline diagnostic procedures but also reduce diagnostic errors and the dependency on extensive clinical testing. Three key features—mean arterial pressure, pulsatile blood pressure index, and resistance-compliance indicator—were found to significantly improve the accuracy of machine learning algorithms in binary heart disease classification. Logistic regression achieved the highest average accuracy among the examined classifiers when utilizing these features. While such novel indicators contribute substantially to the classification process, they should be integrated into a broader diagnostic framework that includes comprehensive patient evaluations and medical expertise. Therefore, the present study offers valuable insights for leveraging data science techniques in the diagnosis and management of cardiovascular diseases.

## 1. Introduction

Cardiovascular diseases are the leading global contributors to mortality, morbidity, and hospitalizations [1]. Among them, ischemic heart diseases have emerged as a particularly severe and complex medical challenge, standing as the foremost cause of death worldwide, according to the World Health Organization [2].

The diagnosis of ischemic heart diseases often relies on intricate and resource-intensive procedures, including comprehensive anamneses and in-depth examination of patient clinical history [3]. This complexity not only increases the cost but also poses additional risks, such as sudden death or chronic health complications.

To confront these complexities, our study introduces resource-efficient machine learning (ML) models that integrate novel, non-invasive clinical indicators known as Campello de Souza (CS) features [4]. Specifically, we require only a tensiometer and a clock for data collection, a simplicity that stands as a strategic advantage, particularly in resource-constrained environments.

The objectives of our study are: (i) to validate the potential of these non-invasive clinical indicators (referred to as CS features) to improve the accuracy of ML-based diagnoses of ischemic heart diseases and (ii) to identify the CS features that have the most significant impact on diagnostic accuracy.

To achieve these objectives, we aspire to make a twofold contribution. First, by proving the effectiveness of CS features, we offer an efficient, patient-friendly, and less resource-intensive method for diagnosing ischemic heart diseases. Second, our research can serve as a practical guide for healthcare professionals aiming to leverage data science for cardiac diagnostics [4].

The structure of the rest of the present article is as follows. Section 2 offers an overview of the introduced CS features. In Section 3, we detail our research methodology, including the ML algorithms employed. In Section 4, the datasets are discussed, and our principal findings are summarized. Section 5 provides our conclusions, limitations, and ideas for future research.

## 2. Biological Indicators

This section begins with a definition of heart disease followed by a description of the biological indicators used for its diagnosis.

### 2.1. Definition of Heart Disease

In the realm of cardiology, heart disease is a broad term that encompasses various conditions affecting the heart, such as coronary artery disease, heart failure, valvular diseases, and arrhythmias. For the purpose of this study, we specifically focus on coronary artery disease, a medical condition that is a subtype of ischemic heart disease. This condition is primarily characterized by reduced blood flow to the myocardium, leading to insufficient oxygen supply to the heart muscle.

Within the context of our study, subjects with coronary artery disease exhibit clinical symptoms of ischemia, such as angina or chest pain, alongside electrocardiogram changes or imaging evidence suggestive of ischemia. These subjects may also have known risk factors such as hypertension, diabetes, or a history of smoking. Conversely, subjects without coronary artery disease do not exhibit these symptoms and have a normal clinical assessment.

It is crucial to clarify that our study involves data from four different subsets, each representing a range of severities specific to coronary artery disease. These subsets contribute to the potential heterogeneity (or variation) in the presentation and diagnosis of the disease. Detailed descriptions of these subsets are provided in Section 4.

### 2.2. Description of Indicators

The indicators discussed here are extensively detailed in [4]. The intent behind their usage is to provide stable, cost-effective, and non-invasive methods that can consistently contribute to heart disease diagnosis.

Classified as indirect, these indicators result from calculations made between measurements directly obtained from the patient. Hence, they represent relationships between the heart rate (HR), systolic blood pressure (SBP), diastolic blood pressure (DBP), and heartbeat period (τ). These measurements can be taken using basic medical tools such as a sphygmomanometer and a stethoscope. The following subsections summarize these indicators.

### 2.3. Mean Arterial Pressure

The mean arterial pressure (MAP) represents the average pressure throughout the cardiac cycle, which spans from [0,τ], with τ indicating a period and HR =1/τ. The MAP is derived from the model stated as

$$\mathrm{MAP}\left(\tau \right)=\frac{1}{\tau }\int_{0}^{\tau }P(t)dt,$$

where P(t) corresponds to the blood pressure in the period *t*. In [4], it was explained that

$$P\left(t\right)=\mathrm{SBP}\times \exp\left(-\frac{t}{\mathrm{RC}}\right),$$

with the RC being equivalent to the product of the peripheral resistance (R) and the compliance (C). Consequently, we have that

$$\mathrm{MAP}\left(\tau \right)=\frac{1}{\tau }\int_{0}^{\tau }\mathrm{SBP}\times \exp\left(-\frac{t}{\mathrm{RC}}\right)dt=\frac{\mathrm{SBP}-\mathrm{DBP}}{\log(\mathrm{SBP})-\log(\mathrm{DBP})}.$$

Clinical relevance: The MAP is often used as a reliable indicator for perfusion adequacy in various clinical settings. It is especially crucial in the management of patients with acute coronary syndromes, where the fine balance between oxygen supply and demand is critical for patient outcomes [5].

### 2.4. Product of Peripheral Resistance and Compliance

As mentioned, the measurement of the RC is derived from the product of R (the peripheral resistance) and C (the compliance) and can be estimated from the first-order approximation [6] given by

(1) $$\frac{dP(t)}{dt}+\frac{P(t)}{\mathrm{RC}}=\frac{1}{C}\sum_{i}\delta \left(t-\tau_{i}\right),$$

where $\tau_{i}=1/{\mathrm{HR}}_{i}$, for i∈{1,2,⋯}, is a sequence of periods of the cardiac cycle. Note that HR${}_{i}$ represents the heart rate corresponding to the *i*-th systolic impulse, and δ is the Dirac delta impulse function [7,8]. The solution to the differential equation formulated in (1) is stated as

$$P\left(t\right)=P\left(0\right)\times \exp\left(-\frac{t}{\mathrm{RC}}\right),$$

with *t* ranging from zero to $\tau_{i}$. Thus, we have that

$$\mathrm{DBP}=\mathrm{SBP}\times \exp\left(-\frac{\tau_{i}}{\mathrm{RC}}\right)=\mathrm{SBP}\times \exp\left(-\frac{1}{\mathrm{RC}}\times \frac{1}{{\mathrm{HR}}_{i}}\right),$$

and then we obtain

$$\mathrm{RC}=\frac{1}{\mathrm{HR}\times \log\left(\mathrm{SBP}/\mathrm{DBP}\right)}.$$

In [9], it was noted that the RC varies throughout the circadian cycle. According to [10], the circadian cycle refers to the 24-hour period in a day primarily due to physiological adjustments in the HR. However, the RC is more stable than the SBP, DBP, and HR, implying that it is less prone to abrupt changes resulting from physiological fluctuations due to daily activities, such as changes in posture. This is attributed to the physiological relationship between the R and C, where they have inversely proportional behaviors. This relationship ensures greater stability in the RC index providing a significant advantage for this index. A higher RC value suggests that if the R or C is high, this indicates an imbalance in the physiological equilibrium between the peripheral resistance and compliance [9].

Clinical relevance: The RC product is instrumental in evaluating the cardiovascular system’s resistance and elasticity. It is particularly relevant for patients with compromised vascular health, such as those with hypertension or atherosclerosis. Maintaining balanced RC values could be indicative of effective treatment strategies [11].

### 2.5. Pulsatile Blood Pressure Index

The pulsatile blood pressure index (PBPI) is a physiological indicator that exhibits stability similar to that of the RC during physiological changes. According to [12], a high PBPI value suggests a malfunction in the cardiovascular regulatory system, leading to variations in the SBP and DBP.

An increase in the PBPI commonly indicates the presence of arterial hypertension, which is a known risk factor for heart disease [3]. The PBPI can be calculated using only the SBP and DBP by means of the expression formulated as

$$\mathrm{PBPI}=\frac{\mathrm{SBP}-\mathrm{DBP}}{\mathrm{DBP}}.$$

Furthermore, the PBPI can be related to the RC, resulting in an indicator denoted as PBPIRC and whose formula is given by

$$\mathrm{PBPIRC}=\frac{\mathrm{PBPI}}{\mathrm{RC}}.$$

According to [12], both high the PBPI and MAP reflect changes in the SBP and DBP, indicating a possible malfunction in the cardiovascular system and the presence of arterial hypertension.

Clinical relevance: The PBPI is a vital risk indicator for cardiovascular diseases, specifically for arterial hypertension. High PBPI levels can be a precursor to hypertension and other cardiovascular conditions, thus making it a tool for early diagnosis and prevention [13].

### 2.6. Harmony Measure

The concept of harmony, introduced in [4] as a harmony measure (HM), draws inspiration from the Kepler harmonic law, which describes the translation movement of planets around the sun in elliptical paths [14]. This law establishes a relationship between the squares of the orbital periods and the cubes of the major semi-axes of the orbits.

In a similar manner, in [4], the HM correlated the cardiac cycle to Earth’s translation cycle. Here, the time τ corresponds to the translation period, the SBP-MAP represents the major semi-axis of the ellipse, and the MAP-DBP denotes the minor semi-axis. In his book, de Souza [4] presented detailed calculations and defined the HM as

$$\mathrm{HM}={\left(\frac{1000}{\mathrm{HR}/60}\right)}^{2}\times \frac{1}{{\left(\mathrm{SBP}-\mathrm{MAP}\right)}^{3}}.$$

Clinical relevance: The HM represents a novel approach to assessing cardiovascular health. While still a subject of ongoing research, preliminary studies have suggested its potential utility in detecting subtle imbalances in the cardiovascular system that may not be evident through conventional indicators [4].

### 2.7. Modeling Ejection Time

**Figure 1:**
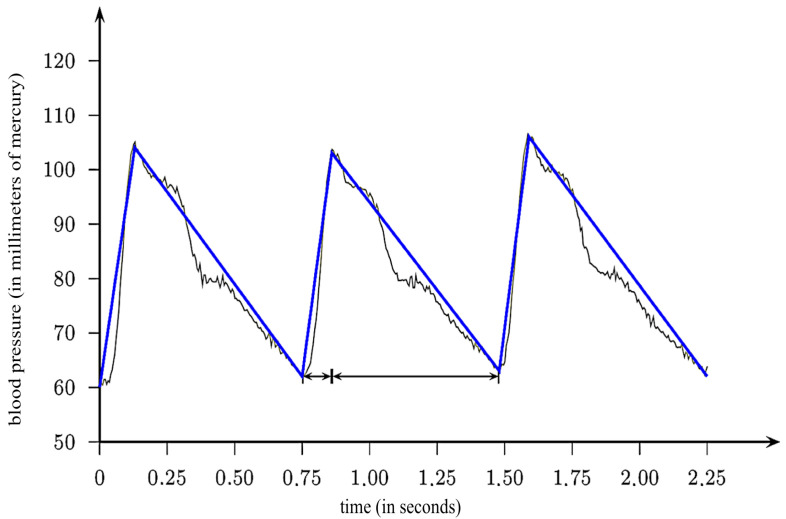
Blood pressure curve outline.

In the triangular pressure wave model, a triangular wave is employed to approximate the blood pressure curve, as illustrated in Figure 1 [15]. Within this model, the MAP is defined as the arithmetic mean between the SBP and DBP.

As outlined in [15], the triangular pressure wave model assumes that the angles of ascent and descent in the pressure curve are equal. This simplification aids in tractability and is common in theoretical modeling. Nevertheless, such assumptions may not fully encapsulate the complexity seen in real-world physiology. This leads to the equation given as

(2) $$\frac{\alpha \tau }{\mathrm{SBP}-\mathrm{DBP}}=\frac{\mathrm{SBP}-\mathrm{DBP}}{(1-\alpha )\tau },$$

where the indicator α represents the proportion of ejection time to the overall cardiac cycle, serving as a critical measure of cardiac performance, and τ represents the heartbeat period as usual. Solving the equation stated in (2) for α, we attain at

$$\alpha =\frac{1}{2}-\frac{1}{2\tau }\sqrt{\tau^{2}-4{\left(\mathrm{SBP}-\mathrm{DBP}\right)}^{2}}.$$

A transformation can further be applied to α to obtain $\alpha_{2}$, reaching

$$\alpha_{2}=\log\left(\frac{1}{\alpha }\right).$$

Clinical relevance: The indicators α and $\alpha_{2}$ offer valuable insights into myocardial contractility and efficiency of the cardiac pump. These indicators could be particularly relevant in specific subsets of patients, such as those with heart failure or myocardial infarction [16].

## 3. ML Techniques

This section provides an explanation of the ML techniques used in this study. We begin by defining the scenarios and then proceed to describe the adapted consistency measure, stages of model selection, classification, and evaluation using performance measures, and conclude by summarizing the methodology in an algorithm.

### 3.1. Datasets and Scenarios

In the analysis conducted using the R computational environment (www.r-project.org, accessed on 13 September 2023), the CS features were included as explanatory variables in the datasets for performing the classification of cardiac patients. Two scenarios were defined for each dataset:

- Scenario 1: It includes the variables V3 (age), V4 (gender), and V11 (history of hypertension); the indicators α, $\alpha_{2}$, HM, MAP, PBPI, PBPIRC, and RC; as well as the response variable *Y*. The variables were selected to prioritize models that utilize low complexity in terms of data collection.
- Scenario 2: It includes the 75 variables in the heart disease directory; the indicators α, $\alpha_{2}$, HM, MAP, PBPI, PBPIRC, and RC; as well as the response variable *Y*.

### 3.2. Adapted Consistency Measure

In this study, we adopt a measure of consistency *d* that calculates the distance between the means of each group considering their respective variances. This measure was proposed in [17] and used in [18] to compare the values of the CS indicators in two groups (cardiac and non-cardiac patients). We determine the relevance of this consistency measure in the classification of individuals with heart disease. Such a measure is defined as

$$d\left(S\right)=\frac{|{\hat{\mu }}_{0}-{\hat{\mu }}_{1}|}{\sqrt{{\hat{\sigma }}_{0}^{2}+{\hat{\sigma }}_{1}^{2}}},$$

where *S* represents the new attribute (CS indicators) inserted into the dataset; ${\hat{\mu }}_{0}$ and ${\hat{\sigma }}_{0}^{2}$ are the estimated mean and variance, respectively, for the CS indicator (*S*) in the group of non-cardiac patients; and ${\hat{\mu }}_{1}$ and ${\hat{\sigma }}_{1}^{2}$ correspond to the estimated mean and variance for the *S* indicator in the group of individuals with cardiac disease.

Each CS indicator is assigned to a calculated value of d(S). It is expected that the indicator with the highest value of d(S) states has the greatest influence on the classification of individuals with cardiac disease.

### 3.3. Features Selection

ML techniques were applied to select the relevant explanatory variables for predicting heart disease, and specific sets of explanatory variables were defined for each model. Within the scope of this study, the defined sets of explanatory variables were determined after undergoing one or more of the selection methods. Then, the selected variables were utilized in the application of different classifiers which are:

- Naive Bayes (NB): This classifier is based on the Bayes theorem, which estimates the probability that an event will occur considering prior information associated with this event [19,20]. The NB method is renowned for its remarkable simplicity and competitive performance compared to other classifiers. However, this method assumes independence between the explanatory variables [21].
- Random forests (RFs): This classifier is an extension of decision trees (DTs), formed by a collection of non-correlated trees. The classification or estimation is determined by a voting process among the trees. DTs are constructed through bootstrapping, where each tree is trained on a different subset of the data [21,22,23]. RFs offer several advantages, including robustness against outliers, low bias, and the ability to capture complex data interactions [21,22].
- Logistic regression (LR): This classifier corresponds to a linear regression in which the response variable is binary. A transformation is applied to ensure the response variable is continuous. Common transformations in the literature include logit, probit, and Cauchy [24,25,26,27]. In this study, the logit transformation was utilized.
- Adaboost: This is an ensemble learning method that combines the results of several weak learning algorithms to generate a more consistent joint response [28]. It iteratively adjusts the weights of misclassified instances to improve the classification performance, focusing especially on difficult instances. Initially, an adjustment takes place, where individuals who did not perform well in the current iteration have greater weight in the subsequent iteration. The classification error rate serves as a measure to evaluate whether this adjustment in weighting improves or worsens the classification. This method tries to enhance the classification performance, particularly for the most challenging individuals to categorize them correctly [29].
- Support vector machines (SVM): This classifier is based on finding an optimal hyperplane that maximally separates the response variable into two classes [30]. The categorization process of the response variable in this classifier utilizes the information from the matrix of explanatory variables to identify an optimal hyperplane. This hyperplane aims to achieve a maximum margin that separates the response into two classes, resulting in an improved classification performance [31]. It is also possible to perform a transformation in the original input space (explanatory matrix). While the maximum margin that distinguishes the classes may be linear in the transformed plane, it can exhibit non-linearity in the original space [32].

### 3.4. Performance Measures

The five model-selection criteria utilized in this study are:

- Information gain (InfoGain): This criterion employs the gain of each explanatory variable using the Shannon entropy [33,34] to select the most significant variables with respect to the response variable [35].
- Variance inflation factor (VIF): This criterion uses the LR model and selects variables using the VIF [36]. The selection obtains a set of variables without collinearity (a strong correlation between two explanatory variables). VIF values greater than 10 indicate strong multicollinearity [37], which affects the estimates of the model [38]. Therefore, variables with a VIF >10 are sequentially removed from the LR with all variables.
- Analysis of variance (ANOVA): This criterion employs ANOVA [39]. Variables that demonstrate statistical significance in ANOVA are chosen, indicating their influence on the response variable.
- ANOVA + VIF: This criterion utilizes ANOVA, followed by an analysis of the VIF. Variables with a VIF greater than 10 are removed from the model.
- Akaike information criterion (AIC): This criterion selects the best model that minimizes its value [40]. The AIC utilizes the model’s likelihood function and the number of explanatory variables in its calculation.
- AIC + VIF: This criterion selects the model by minimizing the AIC, followed by selection based on the VIF (the removal of variables with a VIF greater than 10).

In total, 42 models were selected, encompassing the two scenarios (1 and 2) of datasets and the employed classifiers. Furthermore, the percentages of the times that the CS indicators were deemed relevant by the selection criteria were computed to gain insights into their importance and influence.

To mitigate the risk of overfitting, which arises when a classification method fits well with the training data but performs poorly on unseen data in the testing stage of the classifier, the multiple holdout method [41] with 100 iterations was implemented. This method aimed to obtain the mean and standard deviation (SD) measures of accuracy in the classification of cardiac patients.

In each of the 100 iterations, confusion matrices were generated [42]. An example is shown in Table 1, where true negatives (TNs) correspond to class 0 (non-cardiopaths) correctly classified, false negatives (FNs) represent class 0 (non-cardiopaths) misclassified as 1 (cardiopaths), false positives (FPs) indicate class 1 (cardiopaths) misclassified as 0 (non-cardiopaths), and true positives (TPs) denote class 1 (cardiopaths) correctly classified.

From the confusion matrix [43,44], it is possible to compute the classifier’s accuracy, which is usually expressed as a percentage. The accuracy is calculated as

$$\begin{array}{ccc} \mathrm{Accuracy} & = & \frac{\mathrm{number}\;\mathrm{of}\;\mathrm{individuals}\;\mathrm{that}\;\mathrm{were}\;\mathrm{correctly}\;\mathrm{classified}}{\mathrm{number}\;\mathrm{of}\;\mathrm{total}\;\mathrm{individuals}\;\mathrm{on}\;\mathrm{the}\;\mathrm{sample}}\times 100\\ & = & \frac{\mathrm{TN}+\mathrm{TP}}{\mathrm{TN}+\mathrm{TP}+\mathrm{FN}+\mathrm{FP}}\times 100. \end{array}$$

In the context of a multiple holdout, the estimated average accuracy is given by

$${\hat{\mu }}_{\mathrm{Accuracy}}=\frac{1}{100}\sum_{j=1}^{100}\frac{{\mathrm{TN}}_{j}+{\mathrm{TP}}_{j}}{{\mathrm{TN}}_{j}+{\mathrm{TP}}_{j}+{\mathrm{FN}}_{j}+{\mathrm{FP}}_{j}},$$

where ${\mathrm{TN}}_{j}$, ${\mathrm{FP}}_{j}$, ${\mathrm{FN}}_{j}$, and ${\mathrm{TP}}_{j}$ correspond to the TN, FP, FN, and TP values in the *j*-th iteration of the multiple holdout, respectively.

The estimated SD of the accuracy is stated as

$${\hat{\sigma }}_{\mathrm{Accuracy}}=\sum_{j=1}^{100}\frac{{\left({\mathrm{Accuracy}}_{j}-{\hat{\mu }}_{\mathrm{Accuracy}}\right)}^{2}}{99},$$

where ${\mathrm{Accuracy}}_{j}$ represents the accuracy value calculated in the *j*-th iteration.

Therefore, models that passed the selection criteria involving the VIF and demonstrated a higher average accuracy in the test group were selected for each dataset, considering the dependence between some explanatory variables. Using the selected models, several measures for cardiac prediction performance were calculated as percentages [42,45]. These measures include:

- Average sensitivity (ASe): This is the average percentage of TPs, representing the cardiac patients who were correctly classified within the group of people with heart disease in each iteration of the multiple holdout. This measure is given by
  $$\mathrm{ASe}=\frac{1}{100}\sum_{j=1}^{100}\frac{{\mathrm{TP}}_{j}}{{\mathrm{TP}}_{j}+{\mathrm{FN}}_{j}}.$$
- Average specificity (ASp): This is the average percentage of TNs, representing the non-cardiac patients correctly classified within the group of people without heart disease in each iteration of the multiple holdout. This measure is calculated as
  $$\mathrm{ASp}=\frac{1}{100}\sum_{j=1}^{100}\frac{{\mathrm{TN}}_{j}}{{\mathrm{TN}}_{j}+{\mathrm{FP}}_{j}}.$$
- Average true positive predictive (ATPP): This is the average percentage of true positives in relation to all positive predictions, representing the cardiac patients who were correctly classified within the group of people who were estimated to have heart disease in each iteration of the multiple holdout. This measure is expressed as
  $$\mathrm{ATPP}=\frac{1}{100}\sum_{j=1}^{100}\frac{{\mathrm{TP}}_{j}}{{\mathrm{TP}}_{j}+{\mathrm{FP}}_{j}}.$$

It is expected that the best models among those found in this research are the ones that satisfy the criterion of independence of the explanatory variables and demonstrate higher mean values of accuracy, sensitivity, specificity, and ATPP.

### 3.5. Computational Environment and Conditions

All programs for computational implementation were built and executed using the R software, version 4.2.2 [46,47], on a computer with an Intel (R) Core (TM) i5-5200U CPU 2.20 GHz, 8.00 GB of RAM memory, and an operating system of Windows with 64 bits.

The computational codes for reproducibility of this research can be secured at GitHub repository: https://github.com/Raydonal/Cardiac-Classification (accessed on 13 September 2023).

Table 2 reports the functions used to adjust the models, their respective configurations, and the name of the R packages that contain them. These packages correspond to libraries that present specific functions and data for each type of adjustment [48].

### 3.6. Summary of the Methodology

**Figure 2:**
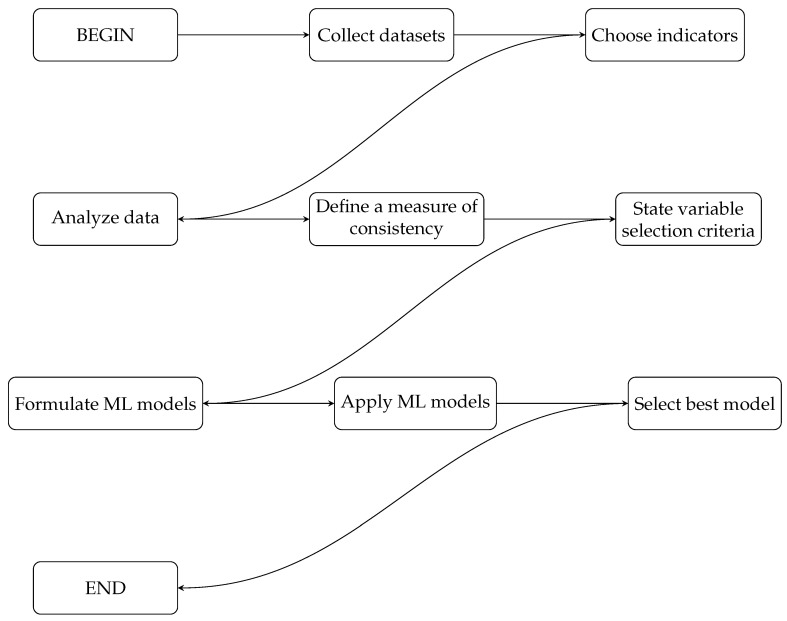
Detailed flowchart of the methodology.

Next, we present a summary of the methodology in Algorithm 1 as well as the corresponding flowchart in Figure 2.
**Algorithm 1** Summary of the methodology using ML techniques to predict cardiac patients.1:Collect datasets with the response variable, the presence of heart disease *Y*, and explanatory variables $V_{j}$, with j∈{1,⋯,r}.2:Choose indicators that may be relevant in the classification of people with heart disease, obtained from the variables in Step 1, as CS indicators.3:Perform a data analysis of the indicators defined in Step 2 and conduct tests of differences between medians.4:Define a measure of consistency to differentiate false signatures of subjects in a dataset as the adapted consistency measure.5:Use techniques to select the relevant explanatory variables in the prediction of heart disease, such as InfoGain, VIF, ANOVA, AIC, ANOVA + VIF, or AIC + VIF.6:Formulate ML classification models, such as Adaboost, LR, NB, RF, and SVR.7:Apply the ML models from Step 6 to classify people with heart disease.8:Select the best ML model using performance measures, considering higher mean values of accuracy, sensitivity, specificity, and ATPP.

## 4. Results and Discussions

This section presents the datasets and results of the computational applications described in Section 3, as well as the discussion and comparison with some findings found in the literature. Initially, the characteristics of the CS indicators are presented for each dataset, followed by the values of the adapted consistency measure. Posteriorly, we discuss which one of the CS indicators was more frequent in the classification of cardiopathy, and the performance of the valid models defined is presented. Given the complexity and urgency of the cardiovascular phenomenon, considerable research continues to be dedicated to the field of this type of diseases [49].

### 4.1. Datasets

The University of California Irvine (UCI) ML data repository (archive.ics.uci.edu, accessed on 13 September 2023), specifically the heart disease dataset [50,51], was utilized for this study. Table 3 provides details of the dataset that contains four subsets based on records from individuals with and without heart disease. The response variable *Y* is the presence of heart disease, with a value of 1 indicating that the individual has heart disease and 0 indicating the absence of heart disease in the subject.

### 4.2. CS Indicators in the Datasets

The application of ML and statistical classification techniques spans various areas of medicine and molecular sciences [52,53,54,55,56,57,58,59,60,61,62]. Numerous studies have been conducted with the aim of identifying factors that can accurately and early on indicate signs of heart disease [63,64]. Building on this extensive body of research, our study focuses on exploring CS indicators using the four key datasets mentioned in Table 3.

Table 4, Table 5, Table 6 and Table 7 present the descriptive statistics of the CS indicators computed using the Cleveland, Hungarian, Long Beach, and Switzerland datasets, respectively. These descriptive statistics include ${\hat{\mu }}_{0}$, ${\hat{m}}_{0}$, and ${\hat{\sigma }}_{0}$, which represent the estimated values for the mean, median, and SD, respectively, of the non-cardiac patient group. Similarly, ${\hat{\mu }}_{1}$, ${\hat{m}}_{1}$, and ${\hat{\sigma }}_{1}$ correspond to the estimated values for the mean, median, and SD, respectively, of the cardiac patient group. These tables also provide the *p*-values of the Wilcoxon–Mann–Whitney test of the difference between medians [65].

Note that the statistics reported in Table 4, Table 5, Table 6 and Table 7 present similar values to those found in [4] on support measures for medical diagnosis. It is also noteworthy that the HM exhibits a high SD in all datasets, while the MAP is more precise. Regarding the applied median difference tests, there was a statistically significant difference in the median MAP values between cardiac and non-cardiac patients, with a *p*-value of 0.0793 in the Cleveland dataset and a *p*-value of 0.0262 in the Hungarian dataset, both suggesting significance at levels of 10% and 5%, respectively. In the Switzerland dataset, none of the new indicators showed statistical significance in the median difference tests, while, in the Long Beach dataset, there was statistical significance at a level of 10% for the HM, with a *p*-value of 0.0886.

Table 8 presents the values of the adapted consistency measures *d*, where a greater relevance is observed for the MAP in the Cleveland dataset (0.1797) and the Hungarian dataset (0.1881), and for the HM in the Long Beach dataset (0.2393) and the Switzerland dataset (0.2568). These results are consistent with the difference of means/medians tests applied to the classes of interest in the research (cardiac and non-cardiac patients).

### 4.3. CS Indicators in the Context of Selected Models

ML applications, such as those discussed above, are particularly valuable in complex diagnostic processes [3]. Moving toward the aim of improving diagnostic accuracy in heart disease, it is crucial to incorporate new indicators, as proposed and analyzed in [4].

Table 9 presents the frequencies at which the selection criteria identified the CS indicators as relevant for the classification of patients with and without cardiac disease. Among the seven indicators described here, the MAP was selected by the criteria in 22 models (52.38%) out of a total of 42, highlighting its significance in the classification of patients with cardiac disease. Additionally, the PBPI was chosen in 13 out of 42 models (30.95%), indicating its importance in the classification process.

To assess model performance, we evaluated the variation in average accuracy across multiple test groups, each pertaining to a different dataset. For example, the Cleveland dataset showed an average accuracy that ranged from 37.70% (±21.31) to 99.20% (±1.17). While such high accuracy rates, such as 99.20%, appear promising, they necessitate cautious interpretation. Specifically, high accuracy can sometimes indicate a risk of overfitting. On the positive side, the relatively low SDs that we observed suggest stable model performance across different data splits. For other datasets, the average accuracies were as follows: from 53.93% (±13.79) to 84.86% (±4.03) for the Hungarian dataset, from 29.78% (±6.59) to 94.28% (±3.47) for the Long Beach dataset, and from 27.41% (±10.49) to 96.01% (±3.25) for the Switzerland dataset. Given these varying performances, it is evident that while our models are promising—especially in terms of stability as indicated by the low SDs—further validation is imperative.

Next, we focus on Scenario 2 to evaluate its models. Overall, as presented in Table 10, the models in this scenario displayed higher average accuracy and lower SDs. For further insights into the variables used, please refer to the following GitHub repository: (https://github.com/Raydonal/Cardiac-Classification) (accessed on 13 September 2023). We identified 12 valid models, based on their VIFs, that stood out for their high average accuracies. Table 11 showcases these 12 models and their performance measures—sensitivity, specificity, and ATPP—on each dataset. We delve into these results by dataset: [Cleveland] Model F with the LR classifier had the highest average accuracy; [Hungarian] Model F with the NB classifier topped the list; [Long Beach] Model E with the LR classifier performed best; and [Switzerland] Model E with the RF classifier was the frontrunner. However, in the case of the Switzerland dataset, there were limitations, where only Model C with the Adaboost classifier showed a non-zero average specificity (5.96%, with an SD of 16.78%). It is essential to clarify our selection criteria for these top-performing models. In this context, the models with the highest average accuracies were chosen based on AIC + VIF criterion, and all featured the variable MAP.

Taking a closer look at the standout models, we find that model F with the LR classifier in the Cleveland dataset exhibited exceptional measures. This model presented an average accuracy of 99.20%, an average sensitivity of 98.23%, an average specificity equal to 100.00%, and an ATPP also equal to 100.00%. Compared to previous studies, these results are highly competitive. For instance, in [66], an accuracy of 89.01%, sensitivity of 80.95%, and specificity of 95.91% were reported. Similarly, in [44], an accuracy of 87.4%, sensitivity of 93%, and specificity of 78.5% were achieved. In [67], an accuracy of 94%, sensitivity of 92%, and specificity of 92.5% were reported. In light of this information, it is evident that our model not only fares well but also suggests advancements in specificity and ATPP.

Considering the classifiers, LR and Adaboost were the methods with the highest accuracy averages, each present in 33.33% of the 12 models selected by the VIF. This high performance can, in part, be attributed to our choice of classification methods, which aligns with the findings in similar studies. For instance, in [63], it was reported that the classifiers Adaboost, DT, and NB obtained the highest accuracy for these datasets, with Adaboost and DT particularly standing out with accuracies ranging from 75.00% to 97.65%.

To further understand the contributing factors to our model’s performance, we delved into an analysis of CS indicators. The relevance of the MAP in the classification of cardiac patients is particularly noteworthy, appearing in almost all the models shown in Table 11. This aligns well with our previous discussions around Table 9 and adapted consistency measures. The variables most frequently appearing in the 12 models (see Table 11) are V4 (gender), present in 11 of these models (91.67%); the MAP, which appears in 8 of the 12 models (66.67%); V61 (distance from the left anterior descending artery), and V40 (exercise-induced ST segment depression with respect to rest, where ST-segment depression is a common electrocardiographic sign of myocardial ischemia during exercise testing), both with relative frequencies of 58.33% each; and V6 (chest pain caused by physical effort), present in half of the models (50.00%). These variables were crucial in the classification of ischemic heart diseases across the diverse subsets discussed earlier in this section. The classifiers we utilized further strengthened the predictive capability of our models.

## 5. Conclusions, Limitations, and Future Research

This section begins with our conclusions about the present study, and then we list its limitations and ideas for further work.

### 5.1. Concluding Remarks

In this study, we set out to improve the classification of patients with coronary artery disease, a subset of ischemic heart diseases, using a diverse range of machine learning algorithms. The use of multiple classifiers, such as Adaboost and logistic regression, not only adds robustness to our study but also gives a comprehensive understanding of the effectiveness of different classification models. What sets our study apart is its resource efficiency. Operating with minimal requirements—just a tensiometer and a clock for data collection—our research proved that significant advancements in ischemic heart disease classification can be achieved with limited resources. This is not a limitation but a unique strength, offering immediate and effective solutions that are particularly crucial in resource-strapped settings. We found the mean arterial pressure to be a pivotal variable, particularly within the Cleveland and Hungarian datasets. This variable was consistently selected among the valid models, bolstering its diagnostic utility. Adaboost and logistic regression emerged as the best classifiers, with this regression proving its high accuracy within the Cleveland dataset.

The insights from this study serve as more than just a stepping stone for future work. They provide immediate and valuable resources to healthcare professionals. Our approach offers a potent blend of efficacy and resource efficiency that can be immediately applied to the diagnosis of ischemic heart diseases, especially in settings where resources are limited. Additionally, the variation in model effectiveness across different datasets hints at the potential for more personalized medical approaches, tailored to the specific demographic, genetic, or environmental characteristics of different population groups.

In summary, this study, built upon a clearly defined scope of ischemic heart diseases, serves as a foundational step in the field of cardiac diagnosis. The classifiers we deployed, including Adaboost and logistic regression, added robustness to our model and were particularly effective across diverse datasets, though further validation is needed for wider applicability. Our resource-efficient methodology offers a viable solution, especially in resource-limited settings, without compromising on diagnostic accuracy. However, we recognize that this simplicity has boundaries, especially when considering the heterogeneity in the presentation and diagnosis of ischemic heart diseases across different population groups.

Our study sets the stage for future research that could add complexity to our models for a more nuanced understanding, while also preserving the essential benefit of minimal resource demands.

### 5.2. Limitations and Future Work

- Simplicity as a strength: A unique selling point of our approach is its resource efficiency, requiring only a tensiometer and a clock for data collection. This is not a limitation, but rather a strategic advantage, especially in resource-constrained environments where quick, yet effective, screenings are essential. In these contexts, the accessibility and ease of use of our model may outweigh the benefits of more complex and resource-intensive methods, offering a viable and immediate solution for diagnosing ischemic heart diseases. That said, we do acknowledge that the model’s simplicity may have boundaries when considering broader applicability. Nonetheless, the focus of this research is on maximizing diagnostic efficacy with minimal resources. Future work will aim to examine the impact of adding more variables and complexity, but the core benefit of our approach lies in its minimal resource demands.
- Hemodynamic indicators: The current study primarily employs hemodynamic indicators as described by Campello de Souza [4]. While effective in the context of ischemic heart diseases, these indicators may also be relevant in diagnosing other cardiovascular conditions, such as heart failure or valvular diseases. Future studies could extend the scope to evaluate such indicators in a wider range of cardiac conditions.
- Disease specificity: Our focus in this study has been primarily on coronary artery disease, which falls under the broader umbrella of ischemic heart disease. While our models have demonstrated effectiveness in this specific context, it is essential to note that heart disease is a broad category that includes various conditions such as heart failure, valvular diseases, and arrhythmias. Future research should explore the applicability of our machine learning models to these other types of heart disease, thereby enriching the diagnostic toolkit available to healthcare professionals.
- Model validation: Our approach already provides a robust baseline due to its simplicity and the diverse set of classifiers that we tested. Nevertheless, future work should engage in more rigorous validation techniques to confirm the generalizability of our models and to mitigate risks such as overfitting.
- Model performance heterogeneity: The variation in model performance across datasets underscores the model’s limitations but also suggests a path for future personalized medicine approaches. We see this not as a limitation but as an opportunity for tailored applications. In particular, the observed heterogeneity in model performance across different datasets raises critical questions about the need for population-specific models in the realm of personalized medicine. We recognize that different models may be more suitable for different populations, and understanding this interaction could lead to a more nuanced, individualized approach to diagnosing ischemic heart diseases. Future research should delve deeper into how our model can be fine-tuned to serve diverse populations effectively.
- Prospective and comparative studies: Our study sets a precedent for resource-efficient diagnostics, but it would benefit from prospective studies comparing its efficacy to that of more resource-intensive methods. This will help confirm its value as a standalone diagnostic tool.
- Future methodologies: To advance in this area, further research employing diverse and larger datasets is required. Studies involving more diverse sample sizes could offer additional insights into the generalizability and reliability of our models.

Further investigations could also explore the integration of personalized medicine approaches to improve the diagnostic accuracy of ischemic heart diseases. Specifically, prospective studies involving larger and more diverse sample sizes could provide critical validation for the models, confirming their efficacy and reliability in clinical settings.

Subsequent research could look at algorithmic enhancements that can make even better use of limited data. Semi-supervised or unsupervised learning techniques could be particularly beneficial in this regard, and statistical improvement in classification methods can be explored based on probability distributions [68,69,70].

## Figures and Tables

**Table 1:** Confusion matrix—example.

Observed Value: *Y*	Estimated Value: $\hat{Y}$	
	**0**	**1**
0	TN	FP
1	FN	TP

**Table 2:** Packages, functions, and their indicators to implement the listed classifier in the R software.

Method	Package	Function	Argument
NB	e1071	naiveBayes	laplace = 0, na.action = na.pass
RF	randomForest	randomForest	ntree = 500, na.action = na.omit
SVM	e1071	svm	scale = F, kernel = “poly”, cost = 100, epsilon = 1.0 × $10^{-12}$, na.action = na.omit
LR	stats	glm	family = binomial (link = “logit”), na.action = na.omit
Adaboost	fastAdaboost	adaboost	nIter = 10

**Table 3:** Heart disease directory—sample size (*n*) and response variable *Y*.

Dataset	*n*	*Y*	
		0	1
Cleveland	282	157	125
Hungarian	294	188	106
Long Beach	200	51	149
Switzerland	123	8	115

**Table 4:** Indicator, estimated mean ($\hat{\mu }$), median ($\hat{m}$), SD ($\hat{\sigma }$), and Wilcoxon–Mann–Whitney *p*-values for the Cleveland dataset.

Indicator	$\hat{\mu_{0}}$	${\hat{\mu }}_{1}$	${\hat{m}}_{0}$	${\hat{m}}_{1}$	${\hat{\sigma }}_{0}$	${\hat{\sigma }}_{1}$	*p*-Value
α	0.0038	0.0040	0.0029	0.0033	0.0029	0.0033	0.4837
$\alpha_{2}$	5.8280	5.7834	5.8284	5.7121	0.7035	0.7473	0.4837
HM	88.2808	79.0296	51.6722	42.4812	118.2997	94.5672	0.3004
MAP	105.1306	108.0998	105.9115	107.8869	11.6754	11.6864	$0.0793{\;}^{*}$
PBPI	0.5439	0.5692	0.5294	0.5556	0.1824	0.1760	0.2434
PBPIRC	19.3680	19.8608	15.7457	17.3187	12.5346	11.7759	0.5273
RC	0.0339	0.0340	0.0329	0.0325	0.0115	0.0113	0.9683

with ${}^{*}$ indicating statistical significance at 10%.

**Table 5:** Indicator, estimated mean ($\hat{\mu }$), median ($\hat{m}$), SD ($\hat{\sigma }$), and Wilcoxon–Mann–Whitney *p*-values for the Hungarian dataset.

Indicator	$\hat{\mu_{0}}$	${\hat{\mu }}_{1}$	${\hat{m}}_{0}$	${\hat{m}}_{1}$	${\hat{\sigma }}_{0}$	${\hat{\sigma }}_{1}$	*p*-Value
α	0.0048	0.0047	0.0039	0.0038	0.0043	0.0035	0.7747
$\alpha_{2}$	5.5739	5.5588	5.5552	5.5628	0.6453	0.6335	0.7747
HM	53.8264	55.4877	38.9873	37.2640	51.6480	77.3438	0.2751
MAP	105.4446	108.6070	102.9850	108.0512	11.4091	12.3450	$0.0262{\;}^{**}$
PBPI	0.5639	0.5971	0.5500	0.5556	0.1502	0.2195	0.1196
PBPIRC	21.7992	22.9755	18.6552	19.2456	13.1868	18.9023	0.6145
RC	0.0295	0.0300	0.0280	0.0291	0.0081	0.0080	0.5268

with ${}^{**}$ indicating statistical significance at 5%.

**Table 6:** Indicator, estimated mean ($\hat{\mu }$), median ($\hat{m}$), SD ($\hat{\sigma }$), and Wilcoxon–Mann–Whitney *p*-values for the Long Beach dataset.

Indicator	$\hat{\mu_{0}}$	${\hat{\mu }}_{1}$	${\hat{m}}_{0}$	${\hat{m}}_{1}$	${\hat{\sigma }}_{0}$	${\hat{\sigma }}_{1}$	*p*-Value
α	0.0038	0.0044	0.0029	0.0039	0.0030	0.0028	0.1033
$\alpha_{2}$	5.8083	5.8785	5.8440	5.5574	0.6754	3.0681	0.1033
HM	67.7813	47.4181	44.0492	34.3783	69.1907	49.5149	$0.0886{\;}^{*}$
MAP	102.7137	106.1425	99.0182	104.4601	13.1111	11.7342	0.1067
PBPI	0.6404	0.6842	0.6085	0.6500	0.2085	0.2005	0.2863
PBPIRC	23.5717	25.9667	20.7905	21.8479	15.9530	13.7833	0.2079
RC	0.0327	0.0299	0.0307	0.0288	0.0106	0.0083	0.1720

with ${}^{*}$ indicating statistical significance at 10%.

**Table 7:** Indicator, estimated mean ($\hat{\mu }$), median ($\hat{m}$), SD ($\hat{\sigma }$), and Wilcoxon–Mann–Whitney *p*-values for the Switzerland dataset.

Indicator	$\hat{\mu_{0}}$	${\hat{\mu }}_{1}$	${\hat{m}}_{0}$	${\hat{m}}_{1}$	${\hat{\sigma }}_{0}$	${\hat{\sigma }}_{1}$	*p*-Value
α	0.0039	0.0038	0.0042	0.0026	0.0031	0.0036	0.9293
$\alpha_{2}$	5.9888	5.9288	5.4949	5.9362	1.1305	0.8388	0.9293
HM	197.6817	112.8445	33.3719	56.5868	285.4129	166.4005	0.9293
MAP	98.9302	104.4039	102.2406	102.0779	16.6840	14.5477	0.5347
PBPI	0.5972	0.5976	0.6587	0.5714	0.2409	0.2411	0.7942
PBPIRC	22.1667	21.8520	26.9187	15.5101	13.9135	19.4161	0.6276
RC	0.0355	0.0358	0.0247	0.0341	0.0161	0.0134	0.5839

**Table 8:** Adapted consistency measures *d* for the listed dataset and indicator.

Dataset	α	$\alpha_{2}$	HM	MAP	PBPI	PBPIRC	RC
Cleveland	0.0648	0.0435	0.0611	0.1797	0.0996	0.0286	0.0083
Hungarian	0.0091	0.0167	0.0179	0.1881	0.1249	0.0510	0.0473
Long Beach	0.1522	0.0223	0.2393	0.1949	0.1514	0.1136	0.2037
Switzerland	0.0204	0.0426	0.2568	0.2473	0.0011	0.0132	0.0163

**Table 9:** Absolute and relative frequencies of indicators in both scenarios.

MAP		PBPI		RC		PBPIRC		HM		α		$\alpha_{2}$		Total
n	**%**	n	%	n	**%**	n	**%**	n	%	n	**%**	n	**%**	
22	52.38	13	30.95	11	26.19	9	21.43	9	21.43	9	21.43	8	19.05	42

**Table 10:** Model and variables in Scenario 2 for the listed dataset.

Model	Variables
Cleveland	
A	V4, V9, V11, V16, V18, V23, V24, V25, V26, V27, V29, V30, V31, V32, V38, V39, V40, V41, V44, V51, V60, V61, V63, V65, V67, V68, V72
B	V3, V4, V9, V11, V12, V14, V15, V16, V18, V19, V23, V24, V25, V26, V27, V29, V31, V32, V33, V34, V35, V38, V40, V41, V43, V44, V51, V59, V60, V61, V63, V65, V67, V68, V71, V72, V73, MAP, PBPI, HM, α
C	V3, V4, V9, V10, V23, V24, V32, V34, V38, V40, V44, V51, V60, V61
D	V4, V10, V34, V40, V44, V51
E	V3, V4, V10, V15, V16, V18, V19, V23, V25, V27, V29, V31, V33, V37, V38, V40, V43, V44, V51, V59, V60, V61, V63, V65, V67, V68, V71, V72, V73, MAP, PBPI, RC, $\alpha_{2}$
F	V3, V4, V15, V16, V18, V19, V23, V25, V27, V29, V38, V40, V43, V44, V51, V60, V61, V63, V65, V67, V68, V71, V72, V73, MAP, PBPI, RC
Hungarian	
A	V4, V5, V6, V7, V9, V11, V16, V24, V25, V26, V27, V32, V38, V39, V40, V41, V72, V73
B	V3, V4, V5, V6, V11, V12, V16, V19, V24, V25, V27, V32, V35, V38, V40, V41, V43, V72 V73, MAP, PBPI, HM
C	V4, V6, V11, V28, V29
D	V4, V6, V11, V28
E	V3, V4, V5, V6, V7, V9, V10, V11, V12, V16, V19, V24, V25, V26, V27, V28, V29, V34, V30, V31, V32, V33, V35, V37, V38, V40, V41, V42, V43, V72, V73, MAP, PBPI, RC, PBPIRC, HM, α, $\alpha_{2}$
F	V4, V5, V6, V12, V16, V19, V24, V27, V31, V32, V34, V35, V38, V40, V41, V42, V72, V73, MAP, RC
Long Beach	
A	V4, V5, V6, V7, V9, V11, V13, V16, V18, V23, V24, V25, V26, V27, V38, V39, V41, V60, V61, V63, V65, V67, V75
B	V4, V5, V6, V7, V11, V12, V14, V15, V19, V28, V59, V60, V62, V63, V64, V65, V68, V70, V71, α
C	V4, V6, V43, V60, V61
D	V3, V4, V5, V6, V7, V10, V11, V12, V13, V14, V15, V16, V18, V19, V28, V29, V31, V32, V33, V37, V38, V39, V40, V42, V43, V59, V60,V61, V62, V63, V65, V66, V67, V68, V70, V71, V72, V73, V74, MAP, PBPI, RC, PBPIRC, HM, α, $\alpha_{2}$
E	V4, V5, V6, V7, V11, V14, V18, V28, V33, V42, V59, V61, V63, V65, V66, V67, V71, V73, MAP, PBPI
Switzerland	
A	V4, V5, V6, V7, V9, V11, V24, V25, V26, V27, V38, V39, V41
B	V4, V7, V25, V27, V33, V38, V39, V40, V59, V61, V62, V65, V67, MAP
C	V7, V61, V67, MAP
D	V4, V6, V7, V19, V24, V25, V27, V32, V33, V36, V38, V39, V40, V60, V61, V62, V64, MAP, PBPI, RC, PBPIRC, HM
E	V4, V7, V19, V27, V33, V38, V39, V40, V61, V64, MAP, HM

**Table 11:** Valid models with higher accuracy averages in Scenario 2 test groups, along with their respective prediction performance measures and CS indicators.

Dataset	Model	# Features	Classifier	Accuracy	ASe	ASp	ATPP	Indicator
Cleveland	B	41	Adaboost	98.58 (1.80)	96.82 (3.93)	99.98 (0.20)	99.97 (0.32)	α, HM, MAP, PBPI
	D	6	LR	81.32 (3.83)	75.76 (7.15)	85.94 (4.53)	81.00 (6.40)	-
	F	27	LR	99.20 (1.17)	98.23 (2.46)	100.00 (0.00)	100.00 (0.00)	MAP, PBPI, RC
Hungarian	B	20	NB	83.10 (4.25)	64.71 (10.51)	93.17 (3.95)	84.39 (7.72)	HM, MAP, PBPI
	D	4	LR	80.84 (3.52)	74.60 (7.16)	84.31 (4.83)	72.15 (7.42)	-
	F	18	NB	83.56 (4.03)	64.16 (10.09)	94.17 (3.52)	86.20 (6.75)	MAP, RC
Long Beach	B	19	Adaboost	79.52 (4.90)	88.00 (4.83)	55.05 (14.21)	85.33 (5.30)	α
	C	5	Adaboost	78.77 (4.54)	88.65 (5.60)	50.55 (16.23)	84.19 (5.76)	-
	E	19	LR	85.47 (6.14)	88.78 (6.16)	74.23 (16.85)	92.22 (5.13)	MAP, PBPI
Switzerland	B	14	RF	93.12 (3.06)	99.33 (1.35)	0.00 (0.00)	93.38 (3.03)	MAP
	C	4	Adaboost	92.52 (3.25)	98.44 (2.18)	5.96 (16.78)	93.74 (3.32)	MAP
	E	12	RF	93.51 (3.25)	99.74 (0.86)	0.00 (0.00)	93.33 (3.08)	HM, MAP

## Data Availability

The R codes and data that support the findings of this study are available here: https://github.com/Raydonal/Cardiac-Classification (accessed on 13 September 2023).
